# Targeting CD117 on hematopoietic stem and progenitor cells impairs CAR T cell activity

**DOI:** 10.1016/j.ymthe.2025.05.033

**Published:** 2025-05-30

**Authors:** Rubina Thomas, Julie K. Ritchey, John F. DiPersio, Miriam Y. Kim

**Affiliations:** 1 Department of Medicine, Division of Oncology, Washington University School of Medicine, Saint Louis, MO 63110, USA

**Keywords:** CAR T cells, CD117, c-kit, HSPCs, AML, lymphodepletion, cell therapy, T cell immunotherapy

## Abstract

CD117 is a cell-surface receptor expressed on hematopoietic stem and progenitor cells and acute myeloid leukemia (AML), and thus CD117-targeting chimeric antigen receptor T cells (CART117) can function as both conditioning for hematopoietic stem cell transplantation and a therapy for AML. We developed human and mouse CART117 to evaluate the safety and feasibility of targeting CD117 in preclinical mouse models. Human CART117 had potent anti-tumor activity while also mediating significant hematopoietic toxicity in a humanized mouse model. Murine CART117 (mCART117) led to systemic and hematopoietic toxicity without anti-leukemic benefit in immunocompetent C57BL/6 mice. Intriguingly, mCART117 was able to eliminate CD117^+^ cells in the spleen but not in the bone marrow (BM). Of note, proliferation of BM CD117^+^ cells in response to lymphodepleting chemotherapy amplified mCART117-mediated systemic toxicity. Alternative lymphodepletion with radiation ameliorated the systemic toxicity of mCART117 but did not improve anti-leukemic efficacy. Immunodeficient mice given mCART117 in the absence of lymphodepletion died from severe pancytopenia, and this effect was recapitulated by regulatory T cell depletion in immunocompetent mice. Increasing CD117 expression on AML improved the anti-leukemic efficacy and toxicity profile of mCART117. In conclusion, mCART117 anti-leukemic activity is impaired in immunocompetent mice when CD117 is expressed at physiological levels on AML.

## Graphical abstract

**Figure:**
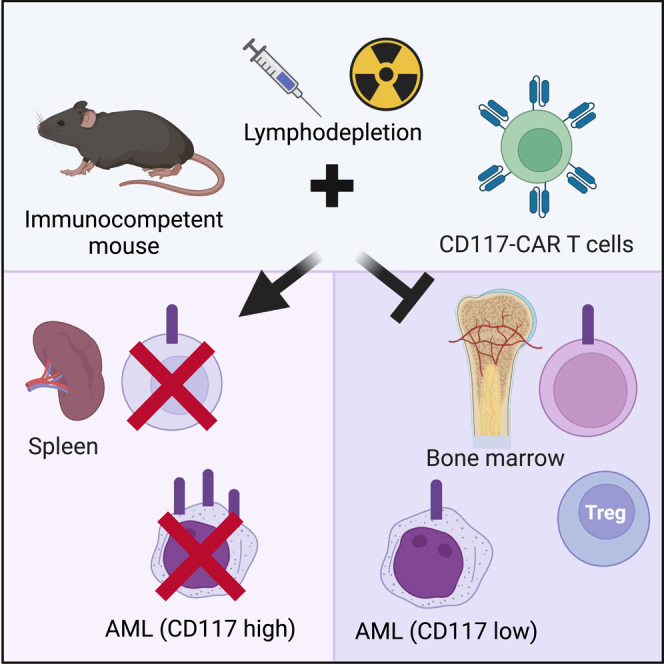

## Teaser

Thomas and colleagues find that CD117-targeting CAR T cells cause systemic and hematopoietic toxicity without anti-leukemic benefit in immunocompetent mice when CD117 is expressed at physiological levels on AML. BM CD117^+^ cell proliferation in response to lymphodepleting chemotherapy amplifies CAR T cell toxicity.

## Introduction

T cell-redirecting therapies such as bispecific antibodies or chimeric antigen receptor (CAR) T cells can successfully treat patients with B cell acute lymphoblastic leukemia. However, acute myeloid leukemia (AML) has had limited success to date with these therapies, despite similar disease kinetics and biodistribution.^1^^,^^2^ One of the primary factors that limit the development of T cell-redirecting therapies in AML is the shared antigen expression between AML and myeloid lineage cells, including hematopoietic stem and progenitor cells (HSPCs), myeloid progenitors, granulocytes, monocytes, macrophages, and dendritic cells. Many of the antigens targeted in AML, such as CD33 and CD123, are expressed across the entire spectrum of myelopoiesis and thus represent a large antigen burden that can impair the anti-tumor efficacy of therapy.

CD117 is an early myeloid antigen that is expressed on HSPCs but not on the majority of mature myeloid cells; therefore, the overall antigen burden is less than that of more established AML targets such as CD33 or CD123. Additionally, CD117-targeting antibodies are being clinically developed as a non-genotoxic conditioning regimen for hematopoietic stem cell transplantation (HSCT).^3^^,^^4^^,^^5^^,^^6^^,^^7^ Using CAR T cells we can achieve both clearance of AML and conditioning for HSCT, which can then be followed with CAR T cell depletion and infusion of allogeneic HSPCs to rescue hematopoiesis, thus streamlining therapy and increasing the chances of long-term disease remission.

To evaluate the feasibility of this strategy, we assessed the activity of human CD117-targeting CAR T cells (hCART117) in xenograft models where we can simultaneously evaluate effects against human tumor and normal hematopoiesis. Additionally, we also evaluated the effects of murine CART117 (mCART117) against murine tumor and hematopoietic cells in an immunocompetent mouse model, to assess the effects of targeting CD117^+^ cells within their native environment, and to address the potential for non-hematopoietic tissue toxicity.

Herein we report that, while hCART117 was effective against both AML and normal hematopoiesis in a human xenograft model, mCART117 was ineffective in immunocompetent mice. We observed systemic and hematopoietic toxicity in mice after mCART117 treatment that greatly exceeded that seen with murine CD19-targeting CAR T cells (mCART19), in part due to HSPC proliferation in response to cyclophosphamide (Cy) lymphodepletion. The toxicity of mCART117 was severe but not lethal. However, despite this toxicity, there was a notable absence of anti-tumor benefit from treatment. Eliminating Cy lymphodepletion improved systemic toxicity but did not rescue the anti-tumor efficacy of mCART117. Regulatory T cell (Treg) depletion enabled effective targeting of HSPCs but exacerbated toxicity of mCART117, resulting in severe pancytopenia and early deaths. These findings recapitulate many of the issues seen in human clinical trials using CAR T cell therapy for AML and provide insights into how these may be overcome to achieve better disease control with decreased toxicity.

## Results

**Figure 1:**
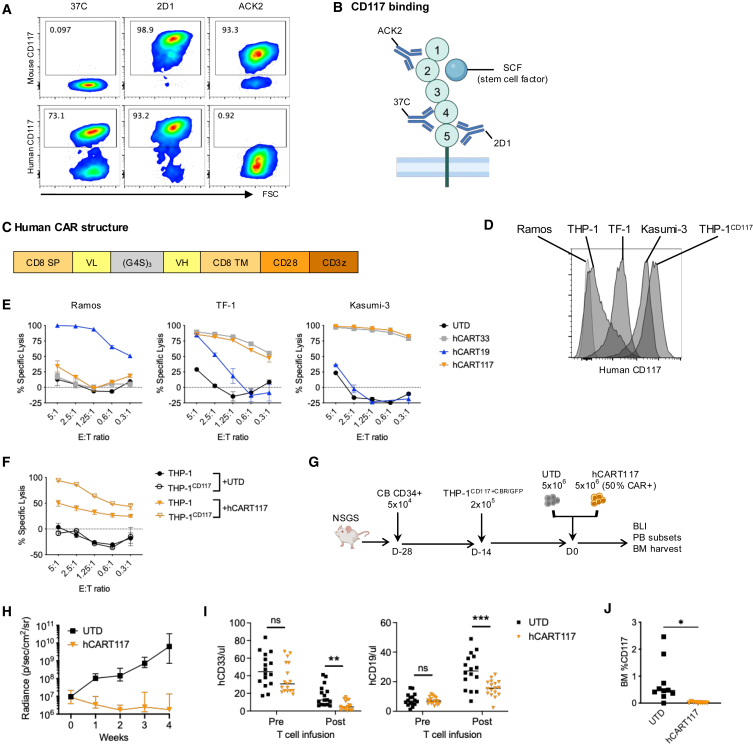
Human CART117 has anti-tumor activity and hematopoietic toxicity in a humanized mouse model (A) Mouse and human CD117 protein binding of the three scFvs used in this paper. (B) Schema of scFv binding sites to CD117 in relation to ligand SCF binding. (C) Structure of the human CAR construct. CD8 SP, CD8 signal peptide; VL, light chain variable region; (G4S)_3_, linker peptide composed of three repeats of four glycines and one serine; VH, heavy chain variable region; CD8 TM, CD8 transmembrane; CD28, costimulatory domain; CD3z, CD3 zeta signaling domain. (D) CD117 expression on the indicated human cell lines. (E) Cytotoxicity of UTD and human CAR T cells targeting CD33 (hCART33), CD19 (hCART19), and CD117 (hCART117) against the indicated cell lines across different E:T ratios. (F) Cytotoxicity of UTD and hCART117 against parent THP-1 and THP-1 engineered to overexpress CD117 (THP-1^CD117^). (G) Experimental schema. NSGS mice were engrafted with human cord blood (CB) CD34^+^ cells to establish human hematopoiesis on day −28, followed by THP-1 cells engineered to express CD117 and CBR/GFP (THP-1^CD117+CBR/GFP^) on day −14. UTD or hCART117 was manufactured from autologous CB T cells and infused on day 0 (*n* = 16–17 per group, three independent experiments). (H) Tumor burden measured by BLI. (I) PB human CD33^+^ myeloid cells and CD19^+^ B cells measured before (pre) and after (post) T cell infusion. (J) BM CD117 expression (percent of total human CD45^+^ cells) in a subset of mice (*n* = 9–10/group). Data are presented as median ± range. Statistical differences between groups were calculated using unpaired Student’s *t* test. ns, not significant. ∗*p* < 0.05, ∗∗*p* < 0.01, ∗∗∗*p* < 0.001.

CD117 has been targeted with a variety of agents including monoclonal antibodies, antibody-drug conjugates, bispecific antibodies and CAR T cells, and many CD117-binding single-chain fragment variable (scFv) sequences are available. We identified three scFvs that are human specific (37C^8^), human and mouse cross reactive (2D1^9^), and mouse specific (ACK2^10^) in their binding to CD117 (Figure 1A). Of note, CD117 has five extracellular immunoglobulin-like domains (D1–D5); the membrane distal domains D1–D3 binds to its ligand stem cell factor (SCF) while the membrane proximal domains D4–D5 dimerize for receptor activation. ACK2 is known to bind to D1–D3 and block ligand binding, while 37C binds to D4/5 and 2D1 binds to D5 (Figure 1B).

We first generated hCART117 using the 37C scFv in a standard CAR structure and lentivirally transduced this into human T cells (Figure 1C). The activity of hCART117 was verified against CD117^+^ human AML cell lines *in vitro* (Figures 1D and 1E). Increasing CD117 expression on THP-1 cells increased their susceptibility to hCART117, indicating that the antigen density of CD117 on target cells affected hCART117 potency (Figure 1F). We then proceeded to evaluate the *in vivo* activity of hCART117 in a humanized mouse xenograft model, where human cord blood CD34^+^ cells were injected into NSGS mice, followed by injection with THP-1^CD117+CBR/GFP^, and either untransduced control T cells (UTD) or hCART117 (Figure 1G). Mice given hCART117 showed decreased tumor burden as measured by bioluminescent imaging (BLI) (Figure 1H). Peripheral blood human CD33^+^ myeloid cells and CD19^+^ B cells were decreased after hCART117 treatment (Figure 1I), and BM CD117^+^ cells were also diminished (Figure 1J). These results corroborate prior studies that have shown that hCART117 can effectively eliminate malignant cells but also leads to significant hematopoietic toxicity.^11^^,^^12^

Of note, the 2D1-based hCART117 was equally effective *in vivo* against Kasumi-3, a human AML cell line with high CD117 expression (Figures S1A and S1B). However, while the human-specific 37C-based hCART117 prolonged survival of NSG mice, the 2D1-based hCART117 reduced survival despite complete tumor clearance (Figure S1C). As CD117 is known to be expressed on HSPCs and also non-hematopoietic cells such as melanocytes, germ cells, and interstitial cells of Cajal, we hypothesized that the deaths after hCART117-2D1 treatment were due to on-target, off-tumor toxicity of targeting CD117 on endogenous mouse cells.

**Figure 2:**
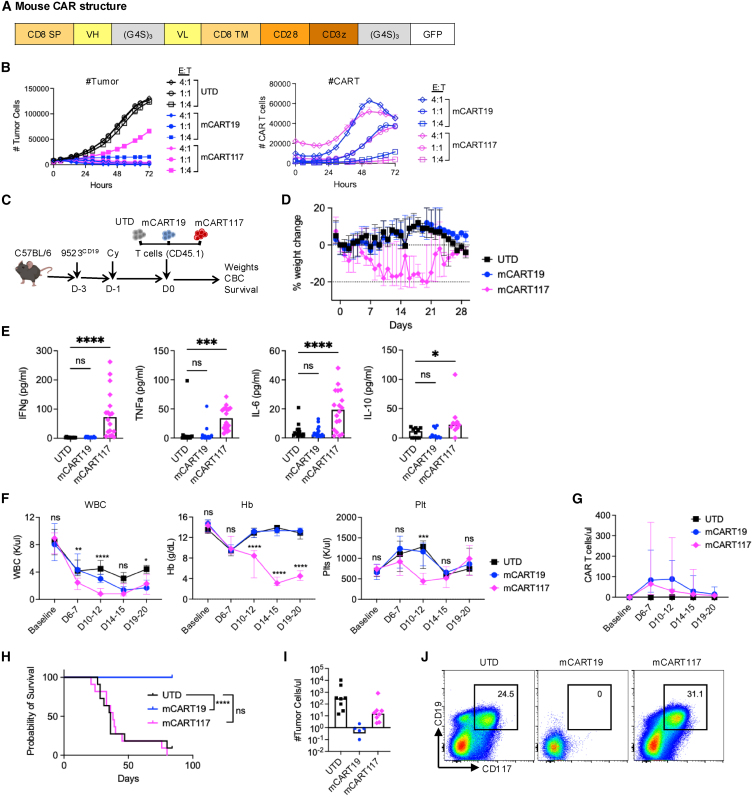
mCART117 has systemic and hematopoietic toxicity without anti-tumor efficacy in immunocompetent mice (A) Structure of the murine CAR construct. (B) The 9523 cell line engineered to express CD19 (9523^CD19^) was plated with CAR T cells at the indicated E:T ratios. RFP^+^ tumor cells and GFP+ CAR T cells were serially quantified using the Incucyte Live-Cell analysis system. (C) Experimental schema: C57BL/6 mice were injected with 1 × 10^6^ 9523^CD19^ i.v. on day −3, followed by Cy 250 mg/kg i.p. on day −1, and 1.5–8 × 10^6^ UTD, mCART19, or mCART117 i.v. on day 0 (*n* = 11 per group, three independent experiments). (D) Serial weight measurements. (E) Plasma cytokine levels on day 7 post-CART treatment. (F) Serial complete blood count (CBC) measurements: WBC, hemoglobin (Hb), and platelet (plts). (G) Peripheral blood (PB) CAR T cell expansion. (H) Overall survival. (I) PB tumor cell numbers at time of death in UTD and mCART117-treated mice, compared with mCART19-treated mice at the same time point. (J) Representative flow cytometry plots gated on tumor cells in each group. Data are presented as median ± range. Statistical differences between groups were calculated using one-way ANOVA with Dunnett’s multiple comparisons test. Kaplan-Meier survival curves were compared using the log rank test. ns, not significant. ∗*p* < 0.05, ∗∗*p* < 0.01, ∗∗∗*p* < 0.001, ∗∗∗∗*p* < 0.0001.

To more stringently evaluate the toxicity of CART117 on endogenous host cells, we generated murine CAR T cells targeting CD117 by cloning the 2D1 and ACK2 scFvs into a CAR structure containing the mouse CD8 transmembrane domain, CD28 co-stimulatory domain, and CD3 zeta signaling domains, followed by GFP to facilitate CAR tracking (Figure 2A). As a control, we also generated mCART19 by cloning the 1D3 scFv into the same CAR structure. All CAR T cells showed similar levels of CAR transduction and CD4/CD8 ratios (Figures S2A and S2B). Our group has also previously generated a murine model of AML by knocking in the human PML-RARα cDNA into the 5′ UTR of the cathepsin G locus in C57BL/6 mice.^13^ A murine AML cell line, 9523, was generated from this model, and was confirmed to express CD117. Both the 2D1 and ACK2-based mCART117 constructs exhibited cytotoxicity against 9523 cells *in vitro* (Figure S2C). The 2D1-based mCART117 was selected for downstream experiments as the efficacy of the scFv was already proven in human CAR T cell experiments, and henceforth mCART117 will refer to the 2D1-based construct unless otherwise stated. To compare the activity of mCART117 directly against mCART19 we transduced 9523 cells with truncated CD19 (9523^CD19^). Both mCART19 and mCART117 showed dose-dependent cytotoxicity and expansion against 9523^CD19^ *in vitro* (Figure 2B).

To assess the activity of mCART117 *in vivo*, we injected 9523^CD19^ cells into C57BL/6 mice, followed by Cy lymphodepletion and either UTD, mCART19, or mCART117 (Figure 2C). Mice receiving mCART117 developed systemic toxicity in the form of weight loss and elevation of plasma cytokines interferon (IFN)-ɣ, tumor necrosis factor (TNF)-⍺, interleukin (IL)-6, and IL-10 (Figures 2D and 2E). Serial complete blood count measurements showed marked anemia after mCART117 treatment and a transient decrease in white blood cells (WBCs) and platelets (Figure 2F). We observed a similar degree of mCART19 and mCART117 expansion in the peripheral blood (Figure 2G). Surprisingly, despite these systemic and hematological toxicities there was no anti-leukemic benefit from mCART117, as the survival was not significantly different from control mice (Figure 2H). We observed high levels of circulating CD19^+^CD117^+^ leukemic cells in mCART117-treated mice immediately before their demise, confirming uncontrolled disease as the cause of death (Figures 2I and 2J).

**Figure 3:**
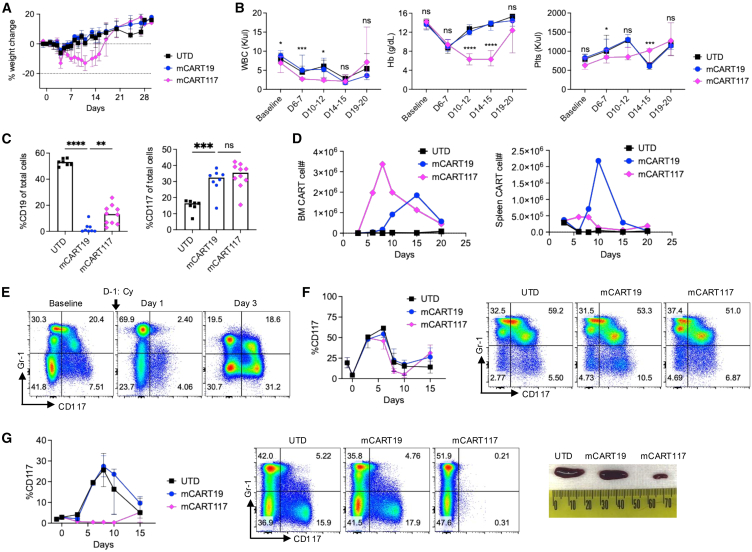
BM CD117^+^ cells expand after lymphodepleting chemotherapy and are unaffected by mCART117 (A–C) C57BL/6 mice were given Cy i.p. on day −1 followed by 1–6 × 10^6^ UTD, mCART19, or mCART117 i.v. on day 0 (*n* = 7 (UTD), *n* = 8 (mCART19), *n* = 10 (mCART117), three independent experiments). (A) Serial weight measurements. (B) Serial CBC measurements. (C) BM CD19 and CD117 expression at day 15. (D–G) BM and spleen were serially harvested from mice in each group and CAR T cells and CD117^+^ cells were quantified by flow cytometry (*n* = 2–5 mice per group at each time point, two independent experiments). (D) Serial quantification of CAR T cell numbers in BM and spleen over time (one representative experiment). (E) Representative flow cytometry plots showing BM CD117 expression at the indicated timepoints in UTD-treated mice. (F) BM CD117 expression over time. Representative flow cytometry plots are shown at day 6 post CART treatment. (G) Spleen CD117 expression over time. Representative flow cytometry plots are shown at day 6 post CART treatment. Picture of spleens at day 10 post CART treatment. Data are presented as median ± range. Statistical differences between groups were calculated using one-way ANOVA with Sidak’s multiple comparisons test. ns, not significant. ∗*p* < 0.05, ∗∗*p* < 0.01, ∗∗∗*p* < 0.001, ∗∗∗∗*p* < 0.0001.

To exclude the possibility of leukemia contributing to the observed toxicities, C57BL/6 mice were given Cy lymphodepletion followed by CAR T cells in the absence of disease. We again observed weight loss and anemia after mCART117 treatment (Figures 3A and 3B). While BM CD19-expressing cells were eliminated by mCART19, BM CD117 expression was not affected in mCART117-treated mice (Figure 3C), despite detectable mCART117 numbers in BM that exceeded that seen for mCART19 (Figure 3D). Notably, we found a striking increase in BM CD117^+^ cells after Cy lymphodepletion in all groups (Figure 3E). The increase in BM CD117 expression coincided with peak mCART117 expansion and was not affected to any degree by mCART117 treatment (Figure 3F). In contrast, CD117^+^ cells were completely eliminated in the spleen, which was accompanied by a smaller spleen size in mice given mCART117 as compared with controls (Figure 3G). These results indicate that BM CD117^+^ cells have unique resistance mechanisms to CAR T cells in immunocompetent mice.

To assess whether these findings are generally applicable to CD117-targeting CAR T cells or unique to the 2D1 scFv used in our mCART117 construct, we also assessed the activity of mCART117-ACK2 *in vivo* using the same experimental schema as Figure 1A. Mice treated with mCART117-ACK2 also exhibited systemic toxicity in the form of weight loss and elevated plasma cytokines (Figures S3A and S3B), while hematopoietic toxicity was less prominent (Figure S3C). Mice treated with mCART117-ACK2 had no survival benefit and no reduction of BM CD117^+^ cell numbers (Figures S3D and S3E). These results indicate that the systemic toxicity coupled with lack of efficacy seen with mCART117 is not unique to the 2D1 scFv.

**Figure 4:**
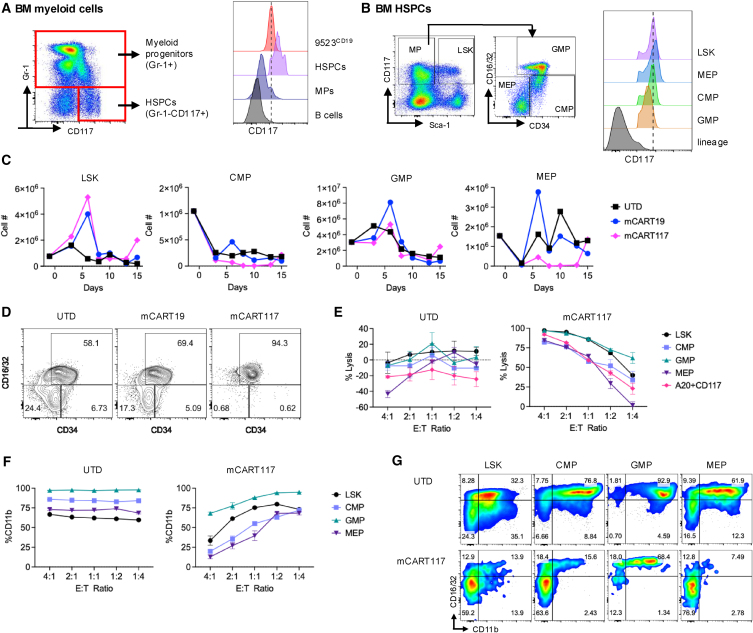
BM HSPCs have differential susceptibility to mCART117 (A) CD117 expression of normal mouse BM HSPCs (CD45^+^CD3^−^CD19^−^Gr1^−^CD117^+^) and myeloid progenitors (CD45+CD3^−^CD19^−^Gr1^+^) in comparison to 9523^CD19^. B cells are shown as CD117-negative controls. (B) CD117 expression of different HSPC populations (gated as shown after excluding lineage-positive cells). (C) Serial quantification of BM HSPC populations over time after CAR T cell treatment. (D) Representative flow cytometry plots are shown at day 8 post CART treatment. (E) *In vitro* cytotoxicity of UTD or mCART117 against sorted BM HSPCs at the indicated E:T ratios. Data are presented as median ± range of technical duplicates. (F) Expression of CD11b on HSPCs by flow cytometry at the indicated E:T ratios. (G) Representative flow cytometry plots after 72-h incubation at an E:T ratio of 4:1.

Of note, CD117 expression in 9523^CD19^ cells was intermediate between HSPCs and myeloid progenitors (Figure 4A). Within the HSPCs CD117 expression was highest in megakaryocyte-erythrocyte progenitors (MEPs) and similar between lineage−Sca1^+^CD117^+^ (LSK) and common myeloid progenitors (CMPs), while slightly decreased in granulocyte-monocyte progenitors (GMP) (Figure 4B). However, the susceptibility of HSPCs to mCART117 did not directly correlate with antigen expression levels, as CMPs and MEPs were more sensitive while LSKs and GMPs were virtually unaffected (Figures 4C and 4D). *In vitro* cytotoxicity of mCART117 against isolated BM HSPC populations did not show any overt difference in susceptibility (Figure 4E). However, expression of CD11b, a marker of myeloid maturation, was decreased in the presence of mCART117 but not UTD (Figures 4F and 4G), suggesting there was preferential cytotoxicity against mature myeloid cells as compared with progenitors.

**Figure 5:**
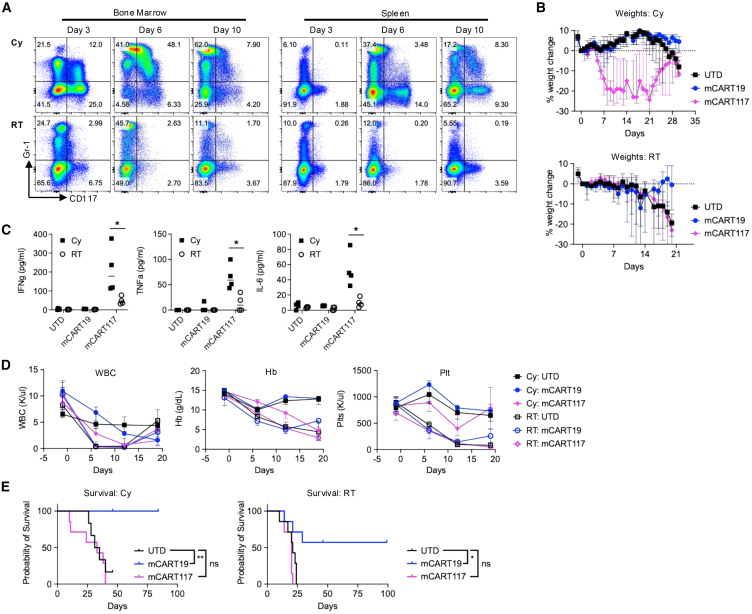
Radiation mitigates systemic toxicity but does not improve hematopoietic toxicity or anti-tumor efficacy of mCART117 C57BL/6 mice were injected with 1 × 10^6^ 9523^CD19^ i.v. on day −4, followed by Cy on day −1 and 1.5 × 10^6^ UTD, mCART19, or mCART117 on day 0. Alternatively, mice were given 6 Gy RT immediately followed by 5 × 10^4^ 9523^CD19^ i.v. on day −1 and 1.5 × 10^6^ T cells on day 0 (*n* = 6–7 per group, two independent experiments) (A) CD117 expression in BM and spleen at the indicated timepoints in UTD-treated mice given either Cy or RT. (B) Serial weight measurements. (C) Plasma cytokine levels on day 6 post-CART treatment. (D) Serial CBC measurements. (E) Overall survival. Data are presented as median ± range. Statistical differences between groups were calculated using unpaired Student’s *t* test. Kaplan-Meier survival curves were compared using the log rank test. ns, not significant. ∗*p* < 0.05, ∗∗*p* < 0.01.

We hypothesized that the Cy-induced myelopoiesis increases toxicity and impairs efficacy of mCART117. Therefore, we proceeded to evaluate total body irradiation (RT) as an alternative method of lymphodepletion that does not increase CD117^+^ cell numbers in BM and spleen (Figure 5A). RT mitigated the early weight loss and cytokine production seen with mCART117 as compared with Cy (Figures 5B and 5C). However, severe pancytopenia was observed in all mice treated with RT, irrespective of CAR T cell treatment (Figure 5D), and again there was no survival benefit with mCART117 (Figure 5E).

**Figure 6:**
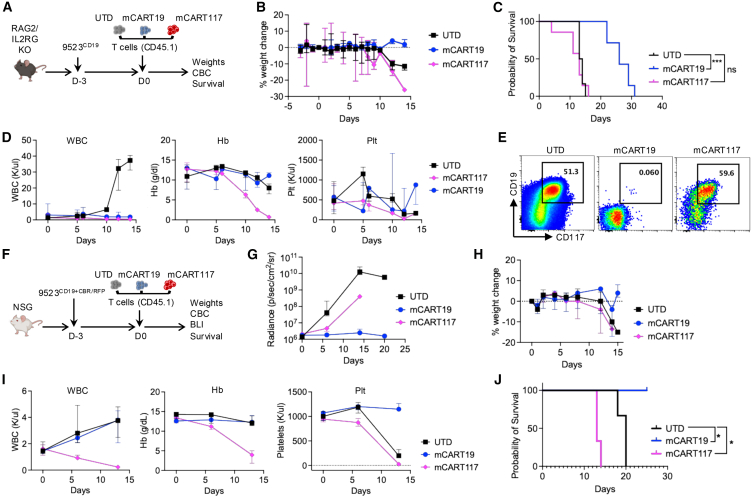
Hematopoietic toxicity of mCART117 is exacerbated in immunodeficient mice (A) Experimental schema. RAG2/IL2RG KO C57BL/6 mice were injected with 5 × 10^5^ 9523^CD19^ cells i.v. on day −3 followed by 5 × 10^5^ UTD, mCART19 or mCART117 i.v. on day 0 (*n* = 6–7 per group, two independent experiments). (B) Serial weight measurements. (C) Overall survival. (D) Serial CBC measurements. (E) Representative flow cytometry plots gated on tumor cells in each group. (F) Experimental schema: NSG mice were injected with 5 × 10^4^ 9523^CD19+CBR/RFP^ cells i.v. on day −3 followed by 4 × 10^6^ UTD, mCART19 or mCART117 i.v. on day 0 (*n* = 5 per group). (G) Tumor burden measured by BLI. (H) Serial weight measurements. (I) Serial CBC measurements. (J) Overall survival. Kaplan-Meier survival curves were compared using the log rank test. ns, not significant, ∗*p* < 0.05, ∗∗∗*p* < 0.001.

To assess the effects of mCART117 in the absence of chemotherapy or radiation, we used mice that are genetically deficient of lymphocytes and so do not require lymphodepletion before CAR T cell treatment. RAG2/IL2RG KO mice were given 9523^CD19^ cells followed by UTD, mCART19, or mCART117 (Figure 6A). RAG2/IL2RG KO mice did not lose weight from mCART117 (Figure 6B), but did develop severe pancytopenia without survival benefit from mCART117 (Figures 6C and 6D). Of note, while leukemic cells were noted in the peripheral blood of mCART117-treated mice at the time of death (Figure 6E), the mice did not exhibit leukocytosis to the extent that we usually observe in mice with terminal leukemia, and so we attribute the etiology of death to severe hematopoietic toxicity rather than AML.

To more closely examine the anti-tumor effects of mCART117 we proceeded to transduce 9523^CD19^ cells with CBR/RFP to enable tracking of tumor burden by BLI. As the black fur of RAG2/IL2RG KO mice impairs BLI signal we used white NOD/SCID/IL2RG^null^ (NSG) mice that are similarly lymphocyte deficient for these experiments. NSG mice were given 9523^CD19+CBR/RFP^ cells followed by CAR T cells (Figure 6F). Serial BLI measurements revealed that mCART117 was effective at reducing tumor burden compared with UTD, albeit not as effective as mCART19 (Figure 6G). While NSG mice also did not exhibit weight loss (Figure 6H), they developed severe pancytopenia and early death, which were again attributed to the hematopoietic toxicity of mCART117 (Figures 6I and 6J).

**Figure 7:**
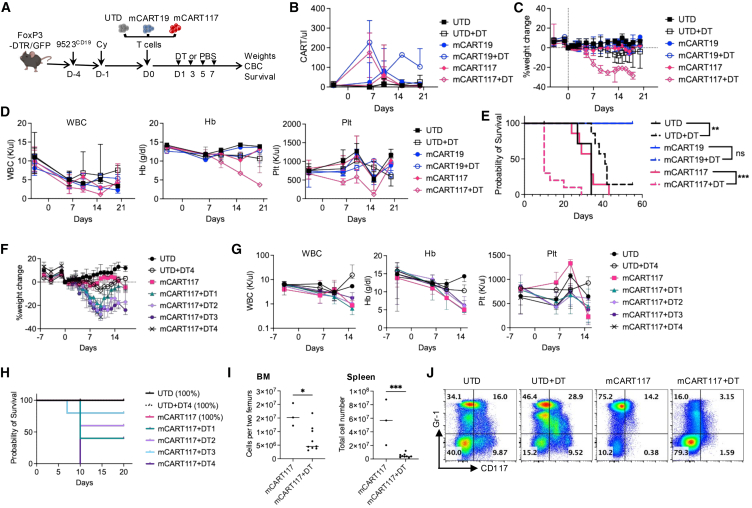
Treg depletion promotes mCART117-mediated killing of HSPCs and early deaths (A) Experimental schema: FoxP3-DTR/GFP mice were injected with 1 × 10^6^ 9523^CD19^ cells i.v. on day −4 followed by Cy i.p. on day −1 and 1 × 10^6^ UTD, mCART19, or mCART117 i.v. on day 0. Mice were then treated with either DT or PBS i.p. on days 1, 3, 5, and 7 (*n* = 8 [UTD, UTD + DT, mCART17], *n* = 4 [mCART19, mCART19 + DT], *n* = 10 [mCART117 + DT], two independent experiments). (B) PB CAR T cell expansion. (C) Serial weight measurements. (D) Serial CBC measurements. (E) Overall survival. (F–H) FoxP3-DTR/GFP mice were given 9523^CD19^ cells and Cy followed by mCART117 and either one (DT1), two (DT2), three (DT3), or four (DT4) doses of DT (*n* = 4–5 per group). (F) Serial weight measurements. (G) Serial CBC measurements. (H) Overall survival. Treatment groups with overlapping survival curves are marked as 100% survival at day 20. (I) BM and spleen cell numbers at days 10–13 after CAR T cell treatment. (J) Flow cytometry plots showing BM CD117 expression at day 10 post CART treatment. Data are presented as median ± range. Statistical differences between groups were calculated using unpaired Student’s *t* test. Kaplan-Meier survival curves were compared using the log rank test. ns, not significant. ∗*p* < 0.05, ∗∗*p* < 0.01, ∗∗∗*p* < 0.001.

The discrepant effects of mCART117 in immunodeficient mice as compared with immunocompetent mice led us to examine the role of endogenous lymphocytes on mCART117 activity. Particularly Tregs have been reported to reside in the BM HSC niche and protect HSCs from immune attack.^14^ We hypothesized that Treg-mediated protection of HSPCs may impair mCART117 activity. To test this hypothesis, we gave FoxP3-DTR/GFP mice 9523^CD19^ cells followed by Cy and CAR T cells, and a subset of mice were given diphtheria toxin (DT) to deplete Tregs (Figure 7A). Of note the CAR T cells in this experiment were manufactured from the FoxP3-DTR/GFP mice to ensure complete Treg depletion from both endogenous and adoptively transferred T cells. Treg depletion increased CAR T cell expansion, which in turn exacerbated mCART117 toxicity and led to early deaths in mice (Figures 7B–7E). Decreasing the duration of Treg depletion did not improve the toxicity profile of mCART117 (Figures 7F–7H). BM and spleen cellularity was markedly decreased with a reduction of myelopoeisis in mice given mCART117 combined with Treg depletion (Figures 7I and 7J).

**Figure 8:**
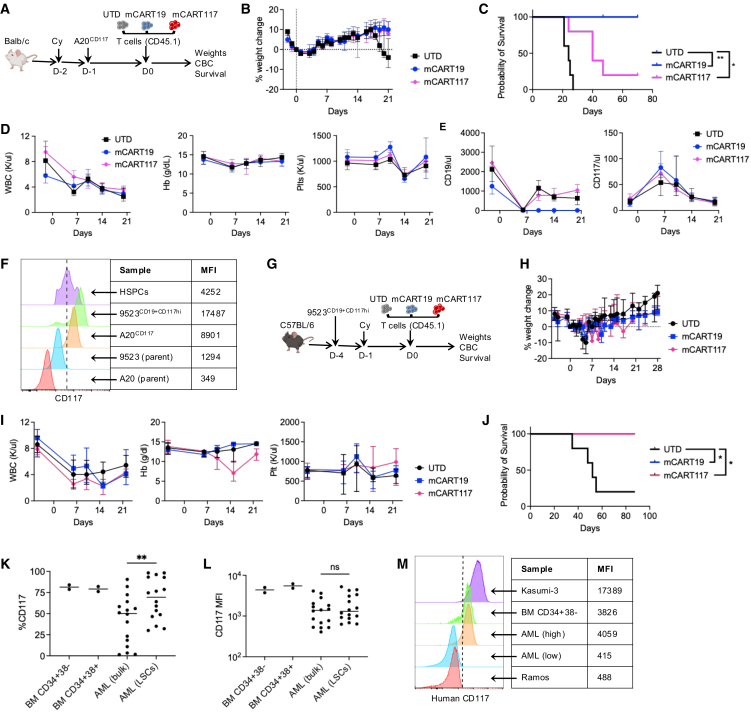
Overexpression of CD117 on target cells decreases toxicity and improves anti-tumor efficacy of mCART117 (A) Experimental schema: Balb/c mice were injected with Cy i.p. on day −2, followed by 5 × 10^5^ A20^CD117^ on day −1, and 1 × 10^6^ UTD, mCART19, or mCART117 i.v. on day 0 (*n* = 5 per group). (B) Serial weight measurements. (C) Overall survival. (D) Serial CBC measurements. (E) PB CD19^+^ and CD117 cell numbers. (F) CD117 expression by flow cytometry on cell lines as compared with BM HSPCs (CD45^+^CD3^−^CD19^−^Gr1^−^CD117^+^). Mean fluorescence intensity (MFI) values of CD117 for each cell type are shown. (G) Experimental schema. C57BL/6 mice were injected with 1 × 10^6^ 9523^CD19+CD117hi^ cells i.v. on day −4, followed by Cy i.p. on day −1 and 1 × 10^6^ UTD, mCART19, or mCART117 i.v. on day 0 (*n* = 5 per group). (H) Serial weight measurements. (I) Serial CBC measurements. (J) Overall survival. (K) Percent CD117 expression of human AML patient samples in bulk (CD45dim) or in LSCs (lineage-CD34+38^−^). Full gating strategy presented in Figure S4. (L) MFI of CD117 expression on human AML patients as compared with normal human BM. (M) CD117 expression with MFI values of BM CD34+38^−^ cells and representative bulk AML samples with high and low CD117 expression as compared with Kasumi-3 and Ramos cell lines. Data are presented as median ± range. Kaplan-Meier survival curves were compared using the log rank test. ∗*p* < 0.05, ∗∗*p* < 0.01.

As an orthogonal model of CAR T cell activity in an immunocompetent host we examined the effects of mCART117 in Balb/c mice (Figure 8A). For these studies we used A20, a murine B cell lymphoma cell line that expresses CD19 at baseline, transduced with truncated CD117 (A20^CD117^) to measure mCART117 anti-tumor activity. Surprisingly, mCART117 exhibited no toxicity and did have anti-tumor benefit against A20^CD117^ cells (Figures 8B–8E). We hypothesized that supraphysiological expression levels of CD117 on malignant cells could mitigate toxicity and improve the anti-tumor efficacy of mCART117, due to preferential targeting of cells with high antigen expression by CAR T cells. To test this hypothesis we transduced 9523^CD19^ cells with truncated CD117, generating 9523^CD19+CD117hi^ cells that were then given to C57BL/6 mice followed by CAR T cell treatment (Figures 8F and 8G). We observed no weight loss, minimal hematopoietic toxicity, and a significant survival benefit from mCART117 treatment against 9523^CD19+CD117hi^ cells (Figures 8H–8J).

Finally, we interrogated the expression of CD117 on human HSPC and AML samples, looking at both bulk disease and the CD34^+^CD38-population enriched for leukemic stem cells (LSCs) (Figures 8K–8M and S4). While there was considerable variation between patients, the median %CD117 expression of CD117 on bulk AML was 50.2%, and this significantly increased in LSCs to 69.4% (Figure 8K). However, the median fluorescence intensity of CD117 did not change between the two populations (Figure 8L), and CD117 expression levels on AML cells were equivalent or lower than BM HSPCs (Figures 8K–8M). These results indicate that the antigen expression profile of CD117 in human AML is not favorable for CAR T cell therapy.

## Discussion

Herein we report that immunocompetent mice treated with mCART117 develop significant systemic and hematopoietic toxicity without anti-leukemic benefit. In contrast with hCART117 that effectively eliminated normal and malignant human CD117^+^ cells in humanized mice, mCART117 was ineffective against murine BM CD117^+^ cells in immunocompetent mice. Mice lacking mature lymphocytes or depleted of Tregs showed greater sensitivity of BM CD117^+^ cells to mCART117, which resulted in severe pancytopenia and early deaths. However, when CD117 was overexpressed on leukemia cells to levels higher than BM HSPCs, mCART117 had clear anti-leukemic effect without significant toxicity.

While human AML has been reported to be resistant to CAR T cell therapy^2^^,^^15^, this was not observed in our murine model, as mCART19 was able to eliminate these same leukemic cells when CD19 was artificially expressed. The striking lack of activity of mCART117 *in vivo* despite cytotoxicity against the same leukemia cells *in vitro* was surprising and initially led us to question the functionality of the CAR construct. However, the 2D1 scFv was effective in a human CAR format while also causing unexpected toxicity in NSG mice. Additionally, mCART117 did eliminate CD117^+^ cells in the spleen, while also producing systemic and hematopoietic toxicity, which would not be expected from a biologically inert product. For these reasons we decided to investigate alternative explanations for why mCART117 did not mediate the desired anti-leukemic effect.

One factor contributing to toxicity was lymphodepleting chemotherapy, which is an essential component of successful CAR T cell therapy in immunocompetent hosts. Cy triggered a massive expansion of BM CD117^+^ cells, presenting a much larger antigen burden for mCART117 than initially expected. Cy is well known to trigger reactive myelopoiesis and is indeed commonly used as a hematopoietic stem cell mobilizing agent in clinical practice, and so human AML-targeting CAR T cells are also likely to encounter an increased antigen burden in patients receiving lymphodepleting chemotherapy. We note that this is a unique challenge for AML that is not an issue for B cell malignancies, as Cy very effectively reduces both normal and malignant CD19^+^ B cells. Unfortunately, while sublethal irradiation as an alternative lymphodepletion did mitigate the systemic toxicity of mCART117, there was no improvement in efficacy. This indicates that, while lymphodepleting chemotherapy presents a problem, more fundamental issues underlie the lack of activity of CAR T cells in AML.

One advantage of our model is that CAR T cells encounter endogenous CD117^+^ cells within their native host environment. The susceptibility of CD117^+^ cells to mCART117 was highly variable depending on tissue location. While CD117 is known to be expressed on melanocytes, we did not observe any pigmentation changes in mice given mCART117. BM CD117^+^ cells were also unaffected, while CD117^+^ cells in the spleen were completely eliminated by mCART117. BM and spleen should be equally accessible to CAR T cells and mCART117 was detected in both organs across serial time points. Therefore, we sought to understand the differential susceptibility of CD117^+^ cells to CAR T cells by interrogating whether cell intrinsic or extrinsic factors were contributing to the resistance of BM HSPCs. As BM is the main site of disease for AML, we surmise that the leukemic cells benefited from the protection provided to BM HSPCs and were shielded from CAR T cell attack, ultimately resulting in the mice dying from disease.

BM HSPCs were found to be more intrinsically resistant to CAR T cells than differentiated myeloid cells, as CD11b^+^ myeloid cells were more effectively targeted by mCART117 *in vitro*. HSPCs are responsible for maintaining hematopoiesis throughout life and thus are designed to survive external injuries that are lethal to other blood cells. Moreover, HSPCs respond to inflammatory stress by proliferating and producing large numbers of myeloid cells to augment host innate defense mechanisms. These intrinsic characteristics of HSPCs can make them particularly difficult to target with CAR T cells.

More strikingly, we found that depleting Tregs improved mCART117 activity against BM HSPCs, as evidenced by pancytopenia and loss of BM CD117^+^ cells. Host Tregs are known to reside in the BM HSPCs niche and provide immune privilege; our data suggest that they can actively suppress CAR T cells targeting HSPCs. The mechanism by which Tregs mediate this function is yet to be determined and will need to be investigated in future studies. Additionally, the BM niche is complex involving multiple cell types and factors, and we do not exclude the possibility that other mechanisms exist that may also contribute to the protection of HSPCs.

Our findings contrast with the reports of CD117-targeting antibodies or antibody-drug conjugates that have been used successfully to deplete BM HSPCs with minimal toxicity in animal models.^3^^,^^5^^,^^16^ These discrepant results can be attributed to the differences in the pharmacokinetic and pharmacodynamic profile of antibodies as compared with CAR T cells. CAR T cells are a living drug that self-propagate and persist long term *in vivo* but are also subject to immunosuppressive mechanisms that are designed to restrict uncontrolled T cell activation. In contrast CD117-targeting antibodies are not affected by the host immunological milieu and are cleared from the body within 4–7 days. The expansion and persistence of CAR T cells are important for their therapeutic efficacy, but in the setting of targeting HSPCs these traits also become liabilities, as they magnify toxicity and impair the ability to perform HSCT rescue in a timely manner.

A prior publication used ACK2-based mCART117 to successfully deplete endogenous HSPCs and achieve donor HSPC engraftment in mice. The apparent discrepancy of our current findings with the prior study can be reconciled when taking into account that the definition of success was quite different in the two studies. The goal of the prior study was to achieve transient reduction of BM CD117^+^ cells at a level sufficient to permit donor HSPC engraftment; in this case, complete elimination of target cells is not necessary, and long-term persistence of mCART117 is not desired. Our goal was to achieve long-term survival from AML, which requires more potent mCART117 activity to eliminate every last CD117^+^ cell. We also note that, in the prior study, even transient BM CD117^+^ cell depletion was only achieved when CXCR4 expression was enforced on the CAR T cells to improve BM trafficking.^17^ We did not pursue this avenue of investigation, as in our model we did not observe impaired mCART117 trafficking to the BM, and furthermore enforcing BM trafficking raises concern for extramedullary relapse of disease in AML.

We used a syngeneic murine AML cell line with native expression of CD117 to avoid the pitfalls associated with artificial overexpression of target antigen on tumor cells. Overexpression of CD117 on malignant cells to levels higher than that of BM HSPCs improved both the toxicity and efficacy profile of mCART117, indicating that a therapeutic window does exist in which mCART117 can be effective as a therapy. Unfortunately, CD117 was only expressed at low levels on the majority of patient AML samples we assessed, and we could not identify any sample that expressed CD117 at a level high enough to ensure effective targeting. Another study evaluating much larger patient sample numbers corroborates our results in that while CD117 expression on AML has a wide variance, the overall expression level is equivalent or lower than normal HSPCs.^18^

The implications of these findings are as follows. First, antigens that are expressed at higher levels on malignant cells as compared with their normal counterparts should be prioritized as targets for therapy, and this can be combined with creative CAR design strategies to further improve specificity.^19^ Second, in the absence of differential expression, methods to preferentially increase antigen expression on AML, or conversely decrease antigen expression on HSPCs, can improve the therapeutic index of CAR T cells.

In this regard, genetic modification of HSPCs to protect them from CAR T cells is an attractive strategy.^20^^,^^21^^,^^22^ CD117 is an essential cell surface receptor for HSPC survival and function and so cannot be deleted without impairing hematopoiesis. However, epitope editing can selectively abrogate CAR T cell binding while still preserving protein function. CD117 epitope editing is already being developed as a method to shield gene-modified HSPCs from CD117-targeting antibody conditioning,^23^ and our results suggest that this could also improve CART117 efficacy.

In summary, we show that CART117 was unexpectedly ineffective against AML despite the development of systemic and hematopoietic toxicity. Clinical trials using CAR T cells in AML have reported frequent development of cytokine release syndrome and yet low rates of disease response, which mirror our results when treating immunocompetent mice with mCART117. This is in contrast with mice given mCART19, where we consistently observe long-term survival without discernable toxicity. Our results highlight the difficulties in using CAR T cells for AML due to the dynamic nature of host HSPC response to therapy, and show that differential antigen expression between normal and malignant cells is key to improving therapeutic outcomes.

## Materials and methods

### CAR constructs

Human CAR19 and CAR33 sequences were derived from the anti-human CD19 scFv FMC63 and anti-human CD33 scFv My96. Human CAR117 was derived from the anti-human CD117 antibody clone 37C (patent US20120189633A1) and clone 2D1 (patent US9932410B2). Each CAR sequence was cloned into a PLVM lentiviral vector containing a second generation CD28-CD3z CAR construct.

The mouse CAR19 sequence was based on the immunoglobulin heavy and light chain sequences of anti-mouse CD19 antibody clone 1D3, followed by the mouse CD8 transmembrane region, mouse CD28 signal transduction domain, and the mouse CD3 cytoplasmic domain. Additionally, to facilitate tracking of CAR transduced cells, GFP was linked to the CAR construct by a glycine-serine linker. This construct was cloned into the SFG retroviral vector.

The mouse CAR117 sequence was obtained from the anti-human CD117 antibody clones ACK2 and 2D1. The immunoglobulin heavy and light chain sequences of ACK2 and 2D1 were cloned into the CAR19 SFG retroviral vector as above. All plasmids were verified by sequencing before virus production.

For the FoxP3-DTR/GFP mouse experiments, the murine CAR constructs were modified to remove GFP and use a truncated human CD34 linked to the CAR via a P2A peptide to enable tracking of the CAR T cells independently of endogenous GFP^+^ Tregs.

### Lentivirus production

Lentivirus for human T cell transduction was generated by transfecting the Lenti-X 293T cells with the PLVM CAR plasmid and packaging plasmids (pRSV.REV, pMD.Lg/pRRE, pMD.G) using Lipofectamine 2000. Virus was harvested 24 and 48 h post transfection and passed through a 0.45-μm vacuum filter to remove cell debris, followed by ultracentrifugation for 90 min at 25,000 rpm at 4°C. Concentrated virus aliquots were stored at −80°C.

### Retrovirus production

Retrovirus for murine T cell transduction was generated by transfecting the Lenti-X 293T cells with the SFG CAR vector and the Ecopac packaging plasmid using Lipofectamine 2000. Virus was harvested 24 and 48 h post transfection, filtered through a 0.45-μm vacuum filter and stored at −80°C without concentration.

### CAR117 binding to CD117

Human and mouse CD117 protein was purchased from SinoBiological (human, 11996-H02H; mouse, 50530-M02H) and labeled using the Abcam Lightning-Link PE conjugation kit (ab102918). CAR-transduced cell lines were stained with human or mouse CD117-PE and binding was assessed by flow cytometry.

### Human CAR T cell production

Human T cells obtained from healthy donors were activated with human CD3/CD28 Dynabeads (Gibco, 40203D) at 3:1 bead to cell ratio and cultured in RPMI complete media (RPMI with 10% fetal bovine serum [FBS], 1% PenStep, 1% Glutamax, 2 mM HEPES, and 50 nM 2-mecaptoethanol). Lentiviral transduction was performed the following day. On days 6–8, beads were removed and CAR expression was evaluated by flow cytometry. T cells were expanded for 10–14 days then frozen and thawed as needed for experiments.

### Mouse CAR T cell production

On day 0, CD45.1^+^ C57BL/6 or BALB/c mice were euthanized and splenocytes were harvested and enriched for T cells using the Miltenyi Pan T cell isolation kit II (#130-095-130) per manufacturer protocol. Purified T cells were activated on the same day of harvest with mouse CD3/CD28 Dynabeads (Gibco, 11453D) at 1:1 bead to cell ratio, and cultured in RPMI complete media supplemented with 10 U/mL IL-2 and 10 ng/mL IL-15. On day 1, T cells were transduced with retroviral supernatant on plates coated with Retronectin (Clontech, T100B) and spinoculated at 2,000g and 30°C for 1 h. On day 3, beads were removed and %GFP was measured by flow cytometry to assess the efficiency of gene transfer. Cells were frozen on days 3–4 and thawed as needed for the experiments.

CAR T cells were generated from FoxP3-DTR/GFP mice using an identical protocol with the exception of using % human CD34 expression to assess CAR transduction.

### Cell lines

Ramos (CRL-1596), THP-1 (TIB-202), TF-1 (CRL-2003), and A20 (TIB-208) cell lines were obtained from ATCC, while Kasumi-3 (ACC 714) was obtained from DSMZ. The 9523 murine acute promyelocytic leukemia (APL) cells were previously generated in our lab by knocking in the human PML-RARa cDNA into the 5′ regulatory sequence of the cathepsin G locus, which produces a high-penetrance APL phenotype in 90% of mice.^13^ Leukemic cells were subsequently immortalized by serial propagation *in vitro*.

Cell lines were mycoplasma tested and antigen expression was confirmed by flow cytometry before use. Lentivirus was used to transduce cells with CBR/GFP, CBR/RFP, human CD117, murine CD19, or murine CD117, followed by single-cell cloning to establish a pure cell population with homogeneous transgene expression.

### Primary human cells

Healthy BM donors and AML patients provided written informed consent before sample collection as part of a study at Washington University School of Medicine. The study was conducted in accordance with the Declaration of Helsinki and approved by the Institutional Review Board and the Human Research Protection Office at the Washington University School of Medicine. Samples were frozen at the time of collection and thawed and stained for flow cytometry according to the gating schema in Figure S4.

### Human CART *in vitro* tumor cytotoxicity assay

Human cell lines expressing CBR/GFP were plated at 5 × 10^4^ cells/well and CAR T cells were added at 5:1 to 0.3:1 ratios in black 96-well flat bottom plates (Thermo Fisher Scientific, 07-200-565) for 24–48 h in technical duplicates. Residual tumor cells were quantified by BLI of the plate after adding 125 μg/mL of D-luciferin (Goldbio, eLUNCA) using an AMI HT optical imaging system (Spectral Instruments). Percent cell lysis was calculated as follows: [1 − (BLI^treated^/BLI^untreated^)] × 100.

### Murine CART *in vitro* tumor cytotoxicity assay

We seeded 9523^CD19+CBR/RFP^ target cells at a density of 10,000 cells per well in a 96-well flat bottom plate in RPMI complete media, and CAR T cells were added to target cells at effector-to-target (E:T) ratios ranging from 4:1 to 1:4. Cytotoxicity was evaluated using the Incucyte Live-Cell Analysis System, in which RFP^+^ tumor cells and GFP^+^ CAR T cells were serially quantified every 6 h up to 72 h using the Incucyte software.

### *In vitro* HSPC cytotoxicity assay

We sorted CD45.2^+^ BM HSPCs according to the gating schema in Figure 4B and seeded at a density of 10,000 cells per well in a 96-well flat bottom plate in IMDM 5% FBS with SCF (25 ng/mL), FLT3L (25 ng/mL), TPO (25 ng/mL), IL-3 (10 ng/mL), and GM-CSF (10 ng/mL). CD45.1^+^ UTD or mCART117 were added to target cells at E:T ratios ranging from 4:1 to 1:4. Cytotoxicity was evaluated using quantitative flow cytometry. Percent cell lysis was calculated as follows: [1 − (cell number^treated^/cell number^untreated^)] × 100. All data points are technical duplicates.

### Mice

The following mice were purchased from Jackson Laboratories: C57BL/6J (#000664), BALB/cJ (#000651), B6.SJL- *Ptprca*^*a*^ *Pepc*^*b*^*/*BoyJ (B6-CD45.1) (#002014), CByJ.SJL(B6)-*Ptprca*^*a*^/J (Balb/c-CD45.1) (#006584), NOD-SCID-IL2Rγ^−/−^ (NSG) (#005557), NOD/SCID/IL2Rgc^null^/CMV-IL3,CSF2,KITLG (NSGS, 013062), and B6.129(Cg)-Foxp3^tm3(Hbegf/GFP)Ayr^/J (FoxP3-DTR/GFP) (#016958). The C57BL/6NTac.Cg-Rag2^tm1Fwa^Il2rg^tm1Wjl^ (RAG2/IL2RG KO) (#4111) mice were purchased from Taconic Biosciences.

All animal studies were approved by the Institutional Animal Care and Use Committee at Washington University (Protocol no. 20180005). Both male and female mice were used for experiments, and similar findings were observed for both sexes. All experimental mice were co-housed within specific pathogen free facilities at Washington University School of Medicine and maintained on *ad libitum* water and standard chow with a 12-h light/dark cycle and a temperature range of 68°F –74°F with 40%–60% humidity. Mice were euthanized if they exhibited signs of illness or discomfort (weight loss of ≥20%, hindlimb paralysis, lethargy, hunched posture), using carbon dioxide asphyxiation followed by cervical dislocation.

### *In vivo* human CAR T cell evaluation

NSGS mice were injected intravenously (i.v.) via lateral tail vein with 5 × 10^4^ human cord blood CD34^+^ cells on day −28. On day −14, we injected 2 × 10^5^ THP1^CD117+CBR/GFP^ cells i.v., and 5 × 10^6^ cord blood-derived CAR T cells were injected on day 0. Tumor growth was measured in mice by intraperitoneal injection of 50 μg/g of D-luciferin (Goldbio, eLUNCA) and BLI using an AMI HT optical imaging system (Spectral Instruments). Images were analyzed using Aura 4.0 In Vivo Imaging Software (Spectral Instruments). Mice were bled via facial vein weekly, and numbers of circulating human leukocyte subsets were evaluated by quantitative flow cytometry. Bone marrow (BM) was harvested at 28–35 days post CAR T cell infusion and human CD117 expression was assessed by flow cytometry.

NSG mice were injected i.v. with 1 × 10^6^ Kasumi-3^CBR/GFP^ cells on day −14 followed by 5 × 10^6^ CAR T cells i.v. on day 0. Tumor burden was serially measured by BLI as above.

### *In vivo* mouse CAR T cell evaluation

C57BL/6 mice were injected i.v. with 1 × 10^6^ 9523^CD19^ cells on day −3 or day −4, followed by intraperitoneal (i.p.) injection of Cy (Cayman, 13849) 250 mg/kg on day −1, then injected i.v. with UTD, mCART19, or mCART117 (manufactured from CD45.1^+^ C57BL/6 mice) cells on day 0. Numbers of T cells injected are indicated in figure legends for each experiment. Mice were bled via facial vein every 4–7 days, and total WBC, hemoglobin, and platelet counts were measured using a Hemavet 950 analyzer (Drew Scientific). Absolute numbers of circulating leukocyte subsets were calculated by multiplying the WBC counts by the frequency of each cell type as measured by flow cytometry. Plasma cytokine levels were measured using the BioLegend LEGENDplex Mouse T Helper Cytokine Panel Version 3 that was customized for the indicated analytes (IFN-γ, TNF-α, IL-6, and IL-10).

Alternatively, C57BL/6 mice were given Cy i.p. followed by UTD, mCART19, or mCART117 in the absence of tumor. Two or five mice from each group were euthanized on days 0, 3, 6, 8, 10, and 15 and BM and spleen were analyzed by flow cytometry to detect CAR T cells, endogenous CD117^+^ cells, and BM HSPC subsets.

Additionally, C57BL/6 mice were given 6 Gy RT immediately followed by 5 × 10^4^ 9523^CD19^ cells i.v. on day −1 and 1.5 × 10^6^ T cells on day 0. RAG2/IL2RG KO mice and NSG mice were given 9523^CD19^ cells i.v. on day −3 followed by T cells on day 0 as indicated in the figure legends. FoxP3-DTR/GFP mice were given 1 × 10^6^ 9523^CD19^ cells on day −4, followed by Cy 250 mg/kg i.p. on day −1, then injected i.v. with 1 × 10^6^ UTD, mCART19 or mCART117 on day 0. Subsequently, mice were given either PBS or DT 10 μg/kg i.p. on days 1, 3, 5, and 7. Balb/c mice were given Cy 250 mg/kg i.p. on day −2 followed by 5 × 10^5^ A20^CD117^ on day −1 and 1 × 10^6^ UTD, mCART19 or mCART117 i.v. on day 0. followed by 5 × 10^5^ mCART19 cells i.v. on day 0. C57BL/6 mice were injected with 1 × 10^6^ 9523^CD19+CD117^ cells i.v. on day −4, followed by Cy i.p. on day −1 and 1 × 10^6^ UTD, mCART19, or mCART117 i.v. on day 0. For all experiments serial WBC counts and leukocyte subsets were measured as above.

### Flow cytometry

For peripheral blood evaluation, 50 μL of fresh blood was added to 2 mL of RBC lysis buffer in flow cytometry tubes and incubated for 10 min at room temperature, followed by a single wash and addition of Fc block (BioLegend #101320), then antibody staining. Spleens were macerated over a 70-μm cell strainer using the piston from a 3-mL syringe to create single cell suspensions, after which 1–2 × 10^6^ cells were transferred to flow cytometry tubes and washed once before Fc block and antibody staining. BM was harvested by flushing femurs for most experiments; for HSPC subset analysis femurs, tibias, pelvis and spine were harvested and crushed using a mortar and pestle to harvest higher cell numbers.

The following antibodies were used for *in vivo* humanized mouse experiments: mouse CD45-APC/Cy7 (BioLegend, 103116, dilution 1:400), human CD45-BV421 (BioLegend, 304032, dilution 1:100), human CD3-PE/Cy7 (Invitrogen, 25-0038-42, dilution 1:200), human CD19-PE (Invitrogen, 12-0199-042, dilution 1:400), human CD33-BV711 (BD Biosciences, 563171, dilution 1:600), and LIVE/DEAD fixable yellow (Thermo Fisher Scientific, L34967, dilution 1:500). BM evaluation was performed using mouse CD45-APC/Cy7 (BioLegend, 103116, dilution 1:400), human CD45-BV421 (BioLegend, 304032, dilution 1:100), human CD117-PE (BD Biosciences, 555714, dilution 1:600), human CD34-APC (BD Biosciences, 561209, dilution 1:400), human CD38-PE/Cy7 (eBioscience, 25-0389-42, dilution 1:400), human lineage-FITC (Invitrogen, 22-7778-72, dilution 1:20), and LIVE/DEAD fixable yellow (Thermo Fisher Scientific, L34967, dilution 1:500).

For *in vivo* mouse experiments the following antibodies were used (all mouse reactive): CD45-APC/Cy7 (BioLegend, 103116, dilution 1:400), Gr1-PE/Cy7 (BioLegend, 108416, dilution 1:800), CD19-PE (BioLegend, 115508, dilution 1:800), CD3-APC (BioLegend, 100236, dilution 1:400), CD117-BV421 (BioLegend, 135124, dilution 1:400), and LIVE/DEAD fixable yellow (Thermo Fisher Scientific, L34967, dilution 1:500).

For BM HSPC analysis, the following antibodies were used (all mouse reactive): Sca1-APC (BioLegend, 108111, dilution 1:400), CD16/32-APC/Cy7 (BD Biosciences, 560541, dilution 1:400), CD117-BV605 (BioLegend, 135120, dilution 1:200), CD34-FITC (Invitrogen, 11-0341-85, dilution 1:100), Lineage-PerCP/Cy5.5 (BD Biosciences, 561317, dilution 1:50), and 7-AAD (BD Pharmingen, 559925, dilution 1:200).

For human BM and AML analysis, the following antibodies were used (all human reactive): CD117-BV421 (BioLegend, 313216, dilution 1:400), CD34-APC (BD Biosciences, 561209, dilution 1:400), CD38-PE/Cy7 (eBioscience, 25-0389-42, dilution 1:400), CD45-BV711 (BD Biosciences, 564357, dilution 1:200), lineage-FITC (Invitrogen, 22-7778-72, dilution 1:20), and LIVE/DEAD fixable yellow (Thermo Fisher Scientific, L34967, dilution 1:500).

All samples were run on an Attune NxT Flow Cytometer (Thermo Fisher Scientific). Data analysis was performed using FlowJo v10.6.1.

### Statistics

All statistical analyses were performed using GraphPad Prism v9 for macOS. Statistical tests used to compare groups are indicated in the figure legends. Significance was defined as a two-tailed *p* value of 0.05 or less.

## Data availability

Data supporting the studies presented in this manuscript will be made available by request directed to the corresponding author (miriamykim@wustl.edu).

## Acknowledgments

This study was supported by an 10.13039/100000054NCI/NIH: K08 CA277000 Award (M.Y.K.) and an 10.13039/100000054NCI/NIH: R35 CA210084 10.13039/100000054NCI Outstanding Investigation Award (J.F.D.).

## Author contributions

R.T.: Investigation, data curation, writing – review and editing. J.K.R.: Investigation, writing – review and editing. J.F.D.: Conceptualization, supervision, writing – review and editing, funding acquisition. M.Y.K.: Conceptualization, methodology, formal analysis, investigation, data curation, visualization, project administration, funding acquisition, writing-original draft, writing – review and editing.

## Declaration of interests

J.F.D. receives research support from NeoImmuneTech, Macrogenics, Wugen, and Bioline; serves as a consultant to Sparc and Vertex; has equity ownership of Wugen and Magenta; serves on the advisory board of Magenta and hC Bioscience; and is an inventor on patent US16/322,803 (Gene editing of CAR-T cells for the treatment of T cell malignancies with chimeric antigen receptors) and US16/401,950 (Compositions comprising an integrin inhibitor and agents which interact with a chemokine and methods of use thereof). M.Y.K. is an inventor on patent US18/467,223 (Methods and compositions for gene editing in hematopoietic stem cells).

## Supplemental information

Document S1. Figures S1–S4

Document S2. Article plus supplemental information
