# Supplementary material for: Targeting CD117 on hematopoietic stem and progenitor cells impairs CAR T cell activity
Source: Mol Ther. 2025 May 30;33(9):4584–99. doi: 10.1016/j.ymthe.2025.05.033 (PMC12432900; doi:10.1016/j.ymthe.2025.05.033)
Supplement: Document S1. Figures S1–S4 [file mmc1.pdf]

YMTHE, Volume *33*

Supplemental Information

Targeting CD117 on hematopoietic stem and progenitor cells impairs CAR T cell activity

Rubina Thomas, Julie K. Ritchey, John F. DiPersio, and Miriam Y. Kim

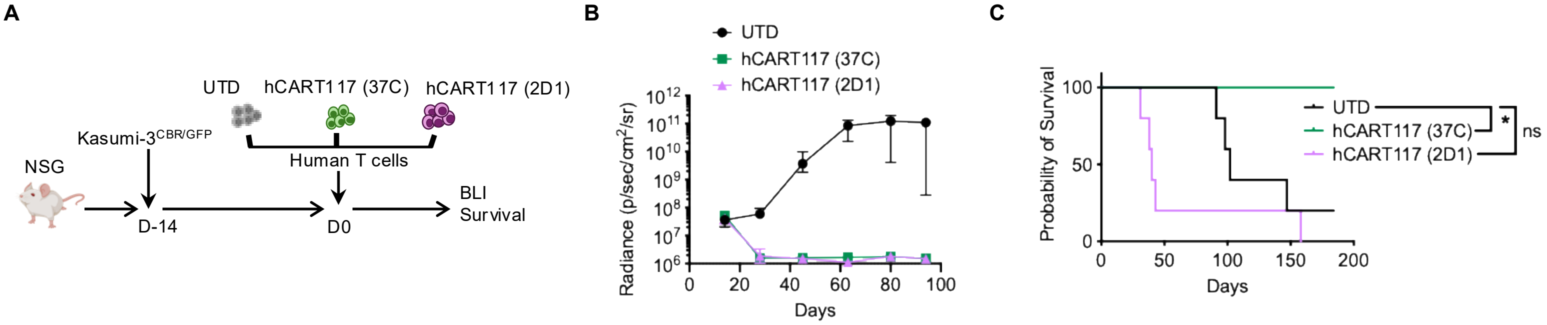

Figure S1. 2D1-based hCART117 leads to early deaths in NSG mice. (A) Experimental schema: NSG mice were injected with 1x10^6 Kasumi-3$^{CBR/GFP}$, a human CD117+ AML cell line, on day -14 followed by 5x10^6 UTD, hCART117 (37C) or hCART117 (2D1) on day 0 (n=5 per group). (B) Tumor burden measured by BLI. (C) Overall survival. Data are presented as median ± range. Kaplan-Meier survival curves were compared using the log-rank test. ns: not significant, *P < 0.05.

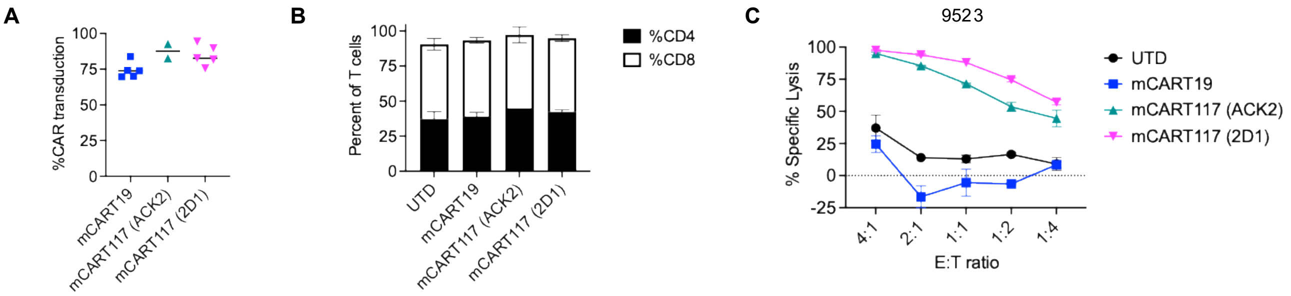

Figure S2. Characterization of murine CAR T cells. (A) CAR transduction as measured by GFP expression. (B) CD4/CD8 expression of untransduced (UTD) T cells, mCART19 and mCART117 (ACK2 and 2D1). (C) In vitro cytotoxicity of the indicated CAR T cells against 9523, a C57BL/6 derived murine AML cell line.

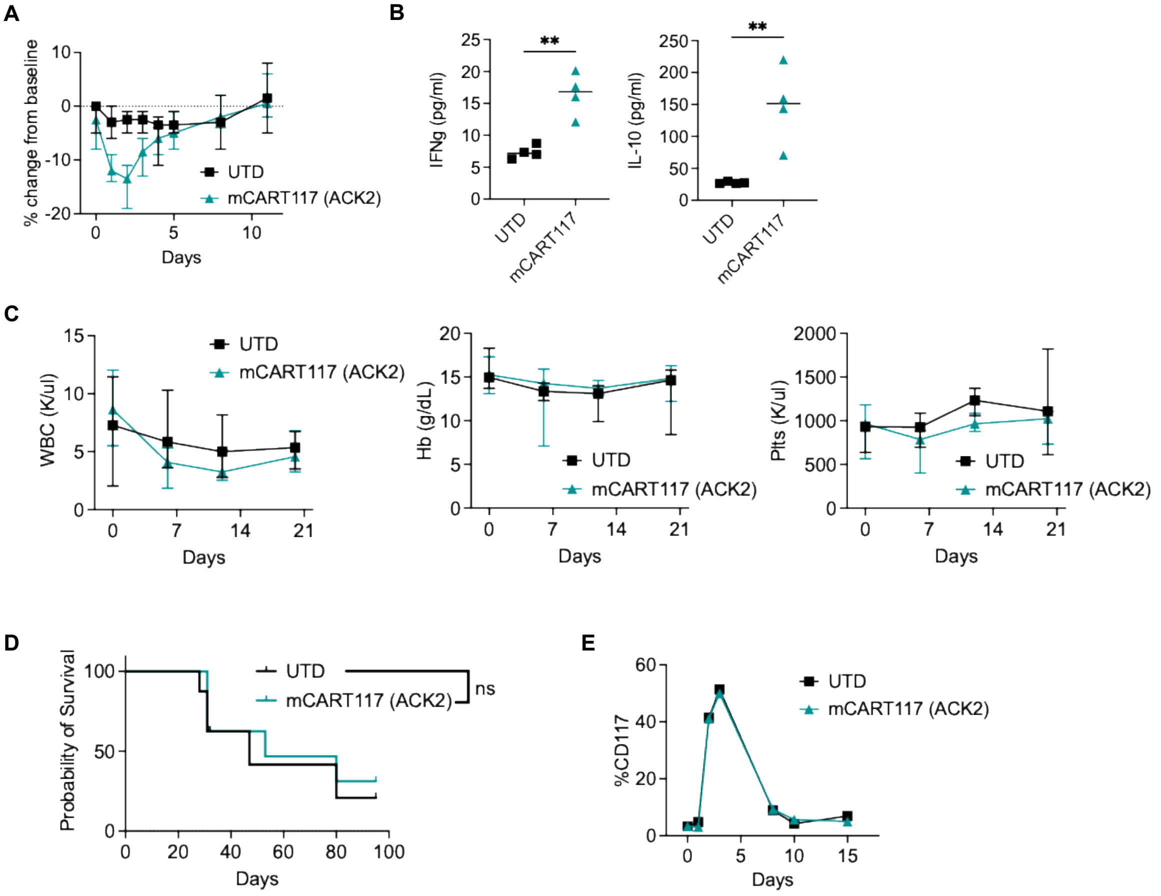

Figure S3. ACK2-based mCART117 causes systemic toxicity without survival benefit or loss of BM CD117+ cells. C57BL/6 mice were injected with $5x10^4$ 9523 cells on day -3, followed by Cy 250mg/kg IP on day -1, and $1x10^7$ UTD or mCART117 (ACK2) on day 0 as in Figure 1A (n=8 per group, two independent experiments). (A) Serial weight measurements. (B) Plasma cytokine levels on day 6 post-CART treatment in a subset of mice (n=4 per group). (C) Serial CBC measurements. (D) Overall survival. (E) BM CD117 expression over time (one mouse per timepoint).

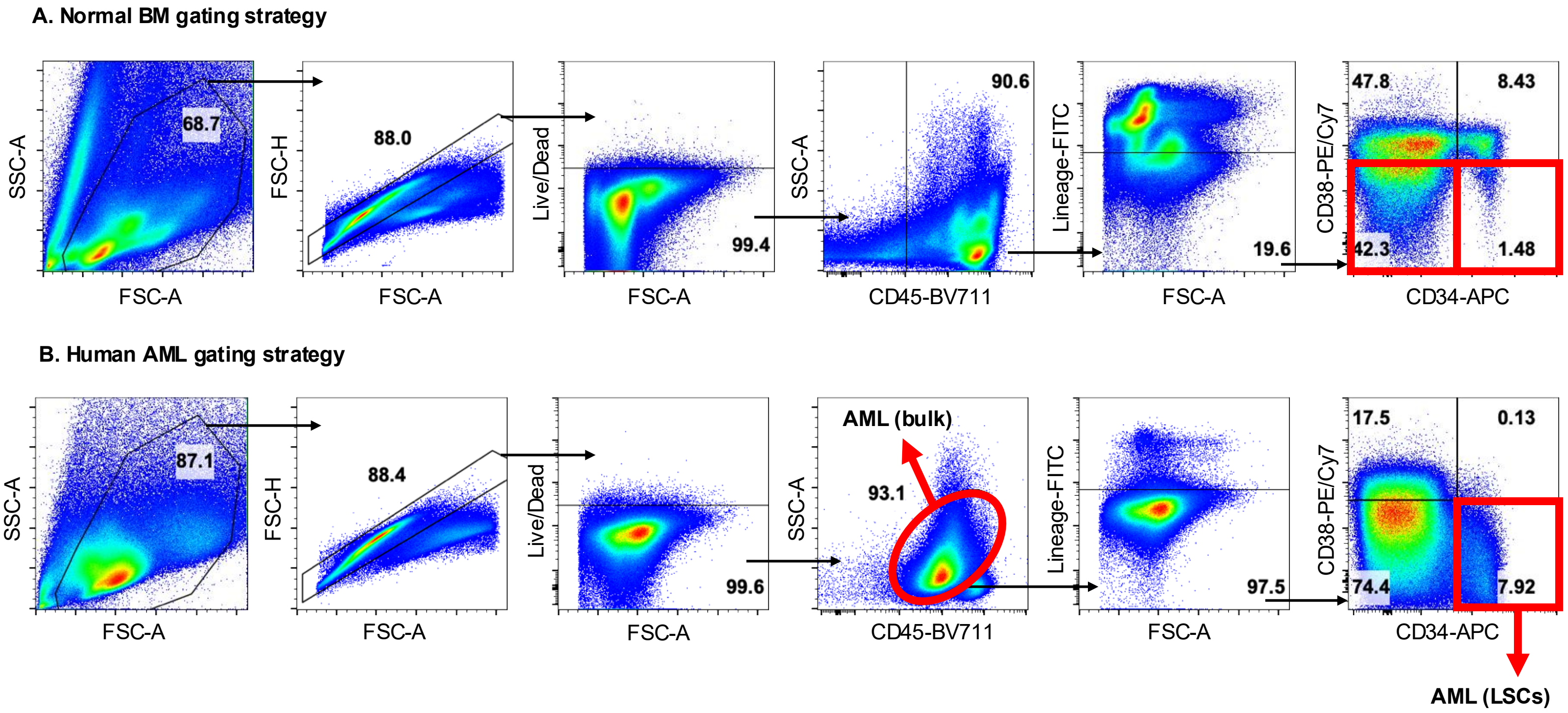

Figure S4. Gating strategy used to identify human BM HSPCs and AML LSCs. (A) Flow cytometry of human BM samples were gated to include live CD45+ lineage-negative cells, followed by evaluation of CD117 expression in CD34+CD38- and CD34+CD38+ cells. (B) Human AML samples were gated to include live CD45dim cells (bulk), or live CD45dim lineage-negative CD34+CD38- cells (LSCs).
